# The Effect of Cue Labeling in Multimedia Learning: Evidence From Eye Tracking

**DOI:** 10.3389/fpsyg.2021.736922

**Published:** 2021-12-16

**Authors:** Jialu Hu, Jinkun Zhang

**Affiliations:** School of Psychology, Fujian Normal University, Fuzhou, China

**Keywords:** cueing, label attribute, multimedia learning, eye movement, spatial contiguity effect

## Abstract

Cue labels are useful during multimedia learning. According to spatial contiguity principle, people learn more when related words and pictures are displayed spatially near one another. Well-arranged labels of multimedia material can greatly facilitate learning. This study used eye tracking to examine the joint influence of label size (large vs. small) and color (included vs. not) on multimedia learning. The results revealed that larger labels led to better retention test performance and a higher AOI glance count, but no cueing effect was found for color. Cues have a certain attention-leading function that promotes the learner remembering the content. These findings suggest that salient labels that provide explanatory information can guide learners’ attention and facilitate learning, though a combination of label size and color salience did not demonstrate a superior cueing effect.

## Introduction

Multimedia learning refers to the psychological construction and processing of speech and picture representation materials (Mayer, 2002; Lawson et al., 2021). With the development of science and technology, multimedia learning has become increasingly popular. A multi-media teaching environment has great potential to improve learners’ learning outcomes. Mayer (2005, 2021) defined multimedia as the co-presentation of words and images. Words refer to the presentation of material in the form of printed text or speech; pictures refer to the presentation of material in the form of images. Examples include static (e.g., illustrations, icons, photos, maps) and dynamic graphics (e.g., animations, videos). Studies have shown that a combination of words and pictures makes it easier for learners to understand scientific explanations than does the single presentation of words (Mayer and Moreno, 2002; Glaser and Schwan, 2015).

It has been determined that a reasonable arrangement of pictures and text can effectively guide learners’ attention and improve the learning effect. To prevent learners’ attention separation (i.e., split attention), researchers have identified the contiguity effect, which relates to how text and pictures are arranged in proximity to one another to improve learning performance (Sweller et al., 1998; Ayres and Sweller, 2014). There are two kinds of contiguity effect: temporal (text and pictures appearing at the same time) and spatial (text and pictures arranged near one another; Mayer, 2001). The spatial contiguity effect was first proposed by Tarmizi and Sweller (1988), who found that when learners solved mathematical problems, information presented in combination reduced learners’ cognitive loads, thus enhancing the learning effect. Mayer (2001, 2021) described this multimedia learning principle as spatial contiguity. When text on a screen is located next to an animation it describes, students learn more deeply than when the text is further away from the corresponding action in the animation (Chandler and Sweller, 1991; Paas and Van Merriënboer, 1994; Moreno and Mayer, 1999; Johnson and Mayer, 2012). The theory is that when the words on a page or screen are close to pictures also appearing there, the learner is better able to establish a psychological connection between the two, whereas when the words are far away, learners must use limited cognitive resources to search for the animation corresponding to the text presented. As a result, learners are less likely to include both in their short-term memory (Mayer, 2005). Previous studies have shown that the learning effect of picture-text contiguity is superior to that of picture-text distance (Ginns, 2006; Holsanova et al., 2009; Mayer, 2021).

At the same time, multimedia learning materials can also have the problem of too much information. In such cases, learners’ attention is attracted to irrelevant information, which consumes learners’ working memory and cognitive resources (Tversky et al., 2002; Lowe, 2003; Ayres and Paas, 2007). Therefore, how to effectively use an instructional design (such as cueing) in multimedia learning materials to enhance the learning effect is a question that has attracted researchers’ attention in recent years (Tabbers et al., 2004; Boucheix et al., 2013).

Cueing is a kind of non-content information (Boucheix and Lowe, 2010) that can attract learners’ attention and promote the selection, organization, and integration of multimedia learning materials. By changing some features of the learning materials in the visual space, cues can lead learners to pay attention to relevant information and integrate old and new knowledge to form a consistent and coherent mental representation (Xie et al., 2016). Cueing can be divided into physical and spatiotemporal cueing, according to different attributes. Physical cueing refers to the physical properties of cueing (such as arrows and colors). Spatiotemporal cueing references the spatial and temporal location properties of cueing (such as progressive animation and random region scaling). This study explores physical cueing. The effects of cueing on multimedia learning are mainly carried out in three steps: guiding learners to notice relevant information, directing learners to organize knowledge, and helping learners to integrate new and existing knowledge. Cueing can have a positive effect on learning. It can help to guide the learner’s attention allocation (Jamet, 2014), improve reaction speed, enhance their understanding of key information, and boost recall, allowing for better knowledge transfer (Luo et al., 2016). Cueing also reduces learners’ cognitive loads, optimizes multimedia learning (de Koning et al., 2010a), and increases the efficiency and effectiveness of information retrieval. A meta-analysis showed that the addition of cues significantly increased retention and transfer performance, as well as the overall fixation time and fixation count in the cue regions (Xie et al., 2016).

A central issue in human cognition is to understand how we encode and maintain a reliable internal representation of the external world. When we look at a scene in the real world, which would typically involve many objects and high levels of complexity, we will later remember only some of the information included in the scene, as a consequence of our limited working memory (WM) capacity (Luck and Vogel, 2013). The internal representation of the external environment is crucial for a number of other cognitive functions, such as long-term memory storage and learning, mental imagery, reasoning, and decisional processes (Fuster, 2006). Previous studies have found that contrast variation and color are the best guides of students’ attention (Wang et al., 2015). In research on scene perception, it is found that “prominent” things can quickly capture attention resources because they have different characteristics from the surrounding environment (Treisman and Gelade, 1980) and can increase the chance of being remembered (Santangelo and Macaluso, 2013; Santangelo, 2015). Participants were found to pay more attention to prominent objects, colors, and events in a scene, and look at them more often and for longer periods of time. In a study of eye movement in response to print ads, readers tended to look first at large font, then at small font, and finally at pictures (Rayner et al., 2001). There are many possible definitions of “saliency,” but all of them converge on this point: something “salient” is something likely to “grab” one’s attentional resources in a bottom-up fashion, and therefore hard to be ignored/unprocessed. The word “salience” derives from roots connoting an assault or sally, which can be interpreted – in this case – upon the senses. The concept of saliency is then strictly connected to bottom-up attention selection, a mechanism which is thought to be primarily driven by capturing (or salient) items/events in the visual scene (Santangelo, 2015). An extensive body of work highlighted the role of visual salience, defined by stimulus intensity, color, orientation, when viewing naturalistic scenes. Salient locations in the image attract subjects’ gaze and attention (Yoshida et al., 2012). Santangelo (2015) found that maximal-saliency targets were better remembered than minimal-saliency targets. Specifically, subjects were faster and more accurate in judging the target location at retrieval when, at encoding, the target object was at the point of maximal saliency compared with the point of minimal saliency. This indicates that bottom-up sensory salience increases the recollection probability and facilitates the access to memory representation at retrieval, respectively.

Previous literature provided evidence that highlight the key role played by low-level sensory features (i.e., line orientation, intensity contrast and color opponency, as indexed by saliency-maps) in biasing attention selection and working memory (WM) encoding (Santangelo, 2015). Fine and Minnery (2009) found that the more salient an icon was, the more accurate subjects were in repositioning the icons. Santangelo and Macaluso found that retrieval accuracy increased along with object saliency at encoding. Overall, this literature consistently demonstrated that bottomup sensory salience increases the probability of an object to be successfully selected, and then stored in memory. This literature highlighted that salient objects (defined in terms of distinguishable features with respect to the surrounding distractors) affect selection priority by capturing attention resources.

Previous research has found that learners spend more time reading text than looking at pictures (van Gog and Scheiter, 2010; Makransky et al., 2019), suggesting that the comprehension process is largely textually directed (Hegarty, 1992; Rayner et al., 2001; Holsanova et al., 2009; Schmidt-Weigand et al., 2010). This has been demonstrated with print ads, newspaper reading, and children’s science text interpretation. The typical pattern of eye movement is that the learner looks at a label before reading the text (Johnson and Mayer, 2012). Labels, which provide explanatory information about the text, help students recall more of the text and generally to perform better (Mayer, 1989). Thus, a key question is: can adding cues to key information in learning materials such as by making labels more visually prominent attract learners’ attention, promote learners’ memory of key information, and thus deepen learning?

In the recent years, eye-tracking technology has been used widely in multimedia learning research because of its convenience in collecting information on visual attention allocation and cognitive processing (Rayner, 2009; Hyönä, 2010). Studies have has confirmed that eye movements reflect visual attention (Li et al., 2019) and what persons fixate on closely relates to what they process, which known as the eye-mind hypothesis (Just and Carpenter, 1976; Ozcelik et al., 2010). In the present study, we employed the eye-tracking method to investigate how cue labeling effects multimedia learning. Based on what has been discussed above, integrating both the font size and the color of label into multimedia learning design might lead to better learning outcomes. More specifically, we anticipate that participants in large label or color condition may significantly outperform those in small label or no-color condition on recognition, retention and transfer tasks, and an interaction effect might occur between variables of font size and color; and participants in the signaling group would show more fixation duration, glance counts, and fixation counts on AOI than non-signaling group.

## Materials and Methods

### Participants

Participants were recruited from Fujian Normal University, because of technical problems with calibration and quality of eye-tracking data, data from six participants were excluded. Seven participants were excluded from data analysis due to their scores on the prior knowledge questionnaire were too high. Finally, 61 students (39 female, *M*_age_ = 21.49 years, *SD*_age_ = 0.34) participated in this study. Sample size was determined using G*Power. Effect size was estimated at 0.4 (based on previous research on cueing effect; see, Xie et al., 2016), α-error probability was set on 0.05, and β-error probability on 0.2. According to this power analysis, a minimum of 52 participants was required. Therefore, the experimental sample size had sufficient statistical test force. According to the results of the pre-test knowledge questionnaire, participants with less than eight points of prior knowledge and experience were randomly assigned to four conditions: 15 in the large label-color group, 15 in the large label-no color group, 15 in the small label-color group, and 16 in the small label-no color group. All participants signed an informed consent form before the experiment, had normal or corrected-to-normal vision, and no achromatopsia.

### Learning Materials

#### Pre-test Questionnaire

The pre-test questionnaire for the experiment was a revised questionnaire on meteorological knowledge. Examples included: “I often read about weather maps,” “I know what a cyclone is,” “I know how cumulonimbus clouds form,” “I know how tornadoes form,” and so on. Responses were given *via* a five-point scale ranging from very little (0) to very much (4). There were six questions in total, with four points possible for each.

#### Learning Materials

Reference high school geography books with a lesson on “tornado formation” were used as the learning materials. Size labels are relative. Here, large labels referred to the font size of the label being larger than the text, and small labels referred to the font size of the label being the same size as the text font. The entire learning process used system control and automatic page turning. Each page’s stay time was 90 s. There was a total of four pages, so the total time was 6 min. Examples are provided below and the red box is the area of interest.

#### Post-test Material

This consisted of recognition, retention, and transfer tests. The recognition test was comprised of five multiple-choice questions, each worth two points, for a total of 10 points. The retention test asked students to describe the formation of a tornado. The correct answer consisted of 24 points, one point for a 1, and 0.5 points for an incomplete score, for a total of 24 points. The transfer test consisted of four questions, including: “What are the characteristics and hazards of tornadoes?” “What are the conditions for tornadoes to form?” “Why do tornadoes ‘suck up’ objects?” and “Why are tornadoes more likely to occur near the ocean than in hot, dry areas?” Two points were possible per question, for a total of eight points.

### Design

This study used a 2 (label: large or small) × 2 (color: included or not) two-factor experimental design. The independent variables were the size and color of the label. Size is relative, so large labels meant the font of the label was larger than the font of the text, and small labels meant that the font of the label was the same as the font of the text. The dependent variables were recognition, retention, transfer, and eye movement. The specific eye movement indicators were total fixation duration of AOI (the sum of all the fixation points in the area of interest; the higher the number, the longer the processing of the area of interest), AOI glance count (the number of times the AOI fixation count jumped to the area of interest from outside the area of interest; the larger the number, the more attention given to the area of interest), and AOI fixation count (the total number of times the area of interest was observed; the higher the number, the more times the participant looked). These three indicators explained the level of attention learners gave to the zone of interest. Generally speaking, the larger the value, the more attention processing was dedicated to the zone of interest.

### Apparatus

The eye movement data acquisition instrument was an Eyelink 1000Plus (SR Research, Canada) desktop eye movement device. The sampling rate was 1,000 Hz, and the screen resolution was 1,024 × 768. Eye movement data were processed using a data viewer. The data were analyzed using SPSS20.0.

### Procedure

Before beginning the experiment, all participants were tested for knowledge of meteorology. The participants selected met the experiment requirements. At the beginning of the experiment, the instructions were presented on a screen: “Welcome to the experiment! You will learn about the formation of tornadoes. There are four pages in total. You have 90 s to learn the information on each page. Once the time expires, the page will automatically turn to the next, and you will not be able to return to reread previous pages. At the end of the session, you will complete a paper-and-pencil test.” The instructions were followed by eye movement calibration. After successful calibration, the formal learning phase began. Learning materials describing how tornadoes form were shown on a computer. At the end of the study period, the participants were given 10-digit addition and subtraction tasks, and then asked to complete the recognition, retention, and transfer tests, in sequence.

### Data Analysis

After each participant completed the experiment, the post-test scores (single choice, free recall, simple questions) were scored by two psychology graduate students who were trained and familiar with the scoring criteria. The consistency coefficient of the two raters for each test score was above 95%. Finally, the data for all participants were entered into SPSS20.0, and a two-factor analysis of variance used for statistical analysis.

## Results

### Learning Outcomes

Table 1 shows the mean scores (and SDs) for the label sizes with and without color levels groups on the recognition test, retention test, and transfer test. To investigate the label sizes with and without color’s effect on multimedia learning outcome, we conducted two factor analysis of variance.

**Figure 1:**
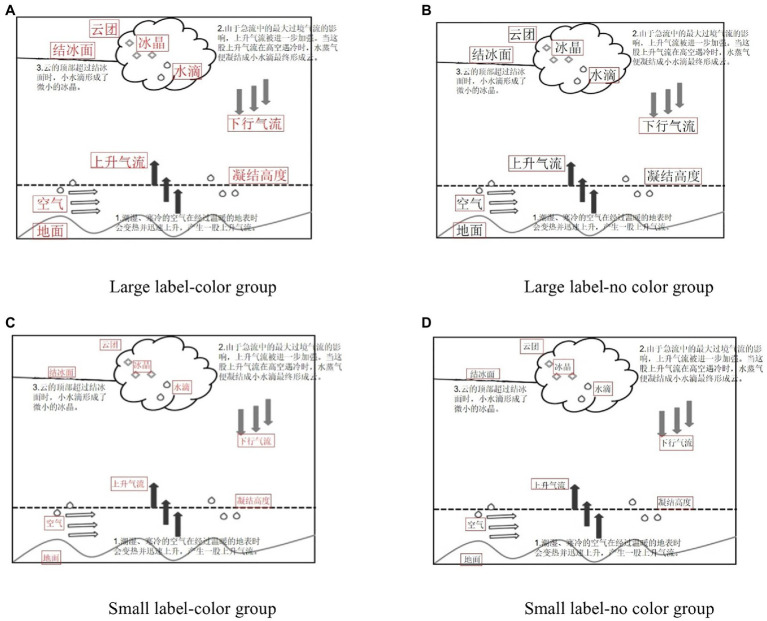
Examples of learning materials. **(A)** Large label-color group. **(B)** Large label-no color group. **(C)** Small label-color group. **(D)** Small label-no color group.

**Table 1:** Scores for label sizes with and without color levels.

	Large		Small	
	Color (*n* = 15)	No-color (*n* = 15)	Color (*n* = 15)	No-color (*n* = 16)
Recognition	7.07(1.98)	7.73(1.67)	6.53(2.67)	7.00(2.53)
Retention	8.13(2.31)	6.83(2.54)	5.40(1.70)	6.03(2.47)
Transfer	4.47(1.36)	4.77(1.12)	4.73(1.59)	4.34(1.06)

In terms of recognition scores, the main effect of label size was not significant, *F* (1,57) = 1.203, *p* > 0.05. There was no significant main effect of color, *F* (1,57) = 0.963, *p* > 0.05. The interaction between label size and the presence or absence of color was not significant, *F* (1,57) = 0.030, *p* > 0.05.

**Figure 2:**
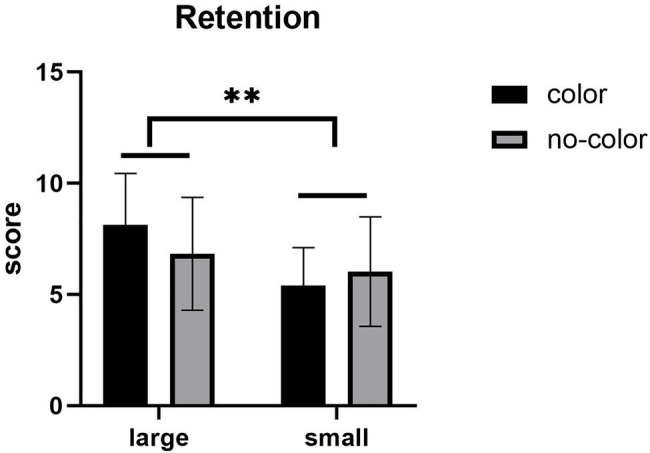
Mean of retention test. Error bars represent standard errors of the means (^**^p < 0.01).

With regards to retention scores, the main effect of label size was significant (see Figure 2), *F* (1,57) = 9.141, *p* = 0.004, *η*^2^ = 0.138, with the retention performance under the large label condition is better than that under the small label condition. There was no significant main effect of color, *F* (1,57) = 0.327, *p* > 0.05. The interaction between label size and presence or absence of color was not significant, *F* (1,57) = 2.728, *p* > 0.05.

For transfer scores, the main effect of label size was not significant, *F* (1,57) = 0.056, *p* > 0.05. There was no significant main effect of color, *F* (1,57) = 0.018, *p* > 0.05. The interaction between label size and presence or absence of color was not significant, *F* (1,57) = 1.082, *p* > 0.05.

### Eye Tracking Outcomes

Table 2 shows the mean scores (and SDs) for the label sizes with and without color levels groups on the fixation duration, glance count, and fixation count. To investigate the label sizes with and without color’s effect on multimedia learning outcome, we conducted two factor analysis of variance.

**Table 2:** Eye movement for different label sizes with and without color levels.

	Large		Small	
	Color (*n* = 15)	No-color (*n* = 15)	Color (*n* = 15)	No-color (*n* = 16)
Fixation duration	32179.73(11085.01)	39522.40(12024.50)	36449.07(10005.81)	26672.25(10680.61)
Glance count	97.20(24.98)	115.87(30.65)	106.13(20.20)	79.69(24.61)
Fixation count	134.53(57.03)	161.80(45.97)	155.40(52.95)	103.69(37.31)

**Figure 3:**
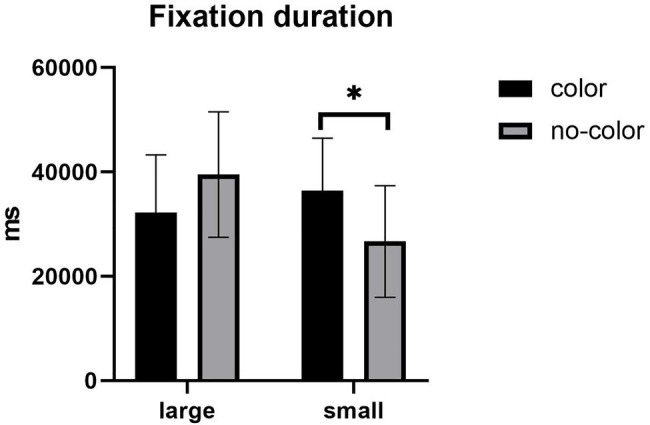
Mean of fixation duration. Error bars represent standard errors of the means (^*^p < 0.05).

In terms of total fixation duration, the main effect of label size was not significant, *F* (1,57) = 2.332, *p* > 0.05. There was no significant main effect of color, *F* (1,57) = 0.188, *p* > 0.05. The interaction between label size and the presence of color was significant (see Figure 3), *F* (1,57) = 9.281, *p* = 0.004, *η*^2^ = 0.140. Further simple effect analyses showed that under the large label condition, there was no significant difference in fixation duration with and without color; under the small label condition, there was a significant difference in the fixation duration with and without color, *F* (1,57) = 6.151, *p* = 0.016. The fixation duration under the color condition was longer than without color. Under the color condition, the fixation duration has no significant difference in the size of the label; under the no-color condition, the fixation duration has a significant difference in the label size, *F* (1,57) = 10.626, *p* = 0.002. The fixation duration under the large label condition was longer than the small label.

**Figure 4:**
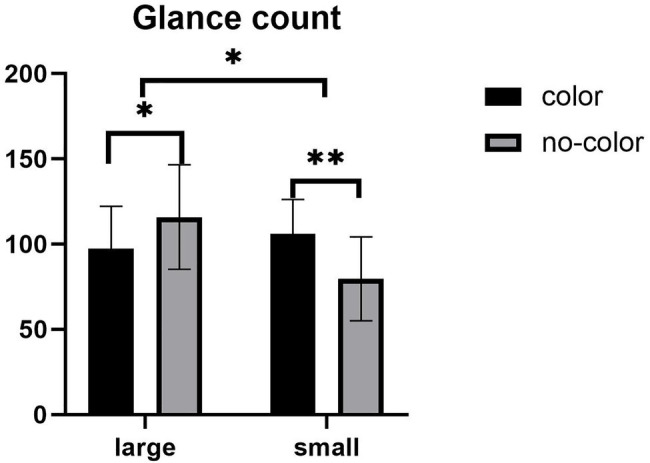
Mean of glance count. Error bars represent standard errors of the means (^*^p < 0.05, ^**^p < 0.01).

With regards to total glance count, the main effect of label size was significant (see Figure 4), *F* (1,57) = 4.394, *p* = 0.041, *η*^2^ = 0.072, with the glance count under the large label condition is more than that under the small label condition. There was no significant main effect of color, *F* (1,57) = 0.358, *p* > 0.05. The interaction between label size and the presence of color was significant (see Figure 4), *F* (1,57) = 12.045, *p* = 0.001, *η*^2^ = 0.174. Further simple effect analyses showed that under the large label condition, there was a significant difference in the glance count with and without color, *F* (1,57) = 4.060, *p* = 0.049. The glance count under the without color condition was more than with color. Under the small label condition, there was a significant difference in the glance count with and without color, *F* (1,57) = 8.412, *p* = 0.005. The glance count under the color condition was higher than without color. Under the color condition, the glance count has no significant difference in the size of the label; under the no-color condition, the glance count has a significant difference in the label size, *F* (1,57) = 15.744, *p* = 0.000. The glance count under the large label condition was higher than the small label.

**Figure 5:**
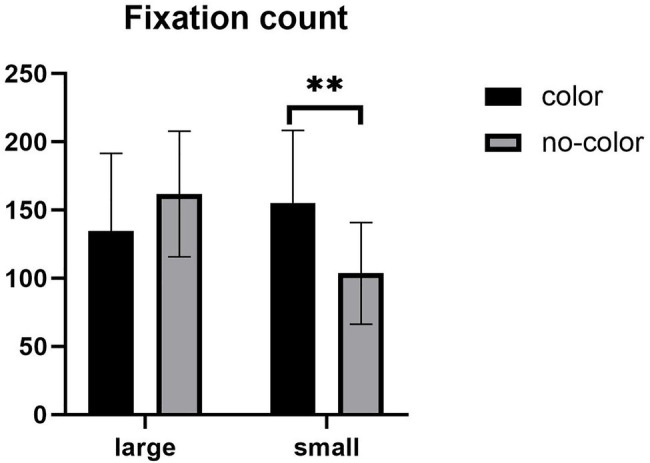
Mean of fixation count. Error bars represent standard errors of the means (^**^p < 0.01).

For the total fixation count, the main effect of label size was not significant, *F* (1,57) = 2.227, *p* > 0.05. There was no significant main effect of color, *F* (1,57) = 0.959, *p* > 0.05. The interaction between label size and the presence of color was significant (see Figure 5), *F* (1,57) = 10.014, *p* = 0.002, *η*^2^ = 0.149. Further simple effect analyses showed that under the large label condition, there was no significant difference in fixation count with and without color. Under the small label condition, there was a significant difference in the fixation count with and without color, *F* (1,57) = 8.725, *p* = 0.005. The fixation count under the color condition was higher than without color. Under the color condition, the fixation count has no significant difference in the size of the label; under the no-color condition, the fixation count has a significant difference in the label size, *F* (1,57) = 11.018, *p* = 0.002. The fixation count under the large label condition was higher than the small label.

## Discussion

This study used an experiment to investigate the effects of different cue-label attributes on learning. The results show that: (1) retention under the large label condition was superior to under the small label condition, and the number of times entering the cue area was greater; (2) The main effect of the presence or absence of color has no significant difference in the post-test scores and eye movement indicators; and (3) On the eye movement index, the interaction between label size and color is significant. Under the condition of a small label, the color plays a better role as a cue; under the condition of no color, the large label plays a better role as a cue.

There was a significant difference in retention scores, but no significant difference in recognition or transfer scores, which is partially consistent with Hypothesis 1. In the eye movement analysis of the cue region, we found that the large labels drew the eye to the region of interest more times than did the small label, which was partially consistent with Hypothesis 2. This shows that cues have a certain attention-guiding function and promote learners’ memorization of learning materials. Bottom-up attention can modulate short-term memory, by increasing the likelihood of attentional “grabbing” items to be remembered later on (Botta et al., 2010).

There was no significant difference in terms of recognition scores, probably because the cues had little effect on relatively simple recognition tasks. These did not require deep processing, just the selection of information appearing in the learning materials. There was no significant difference in transfer scores, perhaps because cues can lead learners to pay attention to the most important content of learning materials, but this does not guarantee that learners will construct and understand that important information accurately (Kriz and Hegarty, 2007). In other words, the mere act of cueing the learner’s attention on a superficial level to the specific location of the learning content does not facilitate deep processing at the conceptual level or establishment of connections between knowledge points (Schnotz and Rasch, 2005; de Koning et al., 2010b). That is to say, not everything that attracts our attention can be successfully coded so that it can be extracted effectively in the future (Santangelo, 2015). Cueing is helpful in facilitating learners’ selection of relevant information, but it does not guarantee learners’ accurate understanding and effective integration of graphics and text information. In addition, in the coding stage, the more effectively an object can obtain attentional resources of participants, the less attentional resources it will have for other objects with low significance (Nardo et al., 2014). In other words, significant label information attracts learners’ attention, reducing learners’ attentional resources for text processing. Therefore, Hypothesis 1 was only partially confirmed. In addition, in the eye movement analysis of the cue area, it was found that the large labels caused the eye to enter the region of interest more times than did the small labels. That is, learners paid more attention to the large labels than they did the small labels, indicating that the cues had a certain attention-leading function. Thus, Hypothesis 2 was also partially verified.

However, combining the two label attributes did not show a better learning effect; that is, no positive effect on learning was found when the color cue was added. This is not consistent with previous studies that have found color cues to promote learning (Kalyuga et al., 1999; Moreno and Abercrombie, 2010; Ozcelik et al., 2010). This inconsistency may be caused by the dynamic nature and complexity of the teaching materials used. Kalyuga et al. (1999) and Moreno and Abercrombie (2010) used animation-based learning materials, which are dynamic. The text used in this study was static. Ozcelik et al. (2009, 2010) not only processed the text as a cue, but also processed the information in the picture that matched the text. This reduced the time required for the learners to locate the relevant elements in the text and images, which may be one reason why the study did not find color cues to facilitate learning.

In terms of eye movement indicators, the interaction between label size and color is significant. Specifically, under the condition of a small label, the color plays a better role as a clue; under the condition of no color, the large label plays a better role as a clue. It may be because when the label becomes larger or colored, the clues on the label are obvious enough to attract the attention of learners. Therefore, the combination of label size and color has not been found to have a better cue advantage.

In addition, Experiment 1 did not guarantee that the learners made full use of the learning time to encode the learning material. In other words, the time given for encoding does not always equal the presentation time of the learning item (Kriz and Hegarty, 2007). The participants may have finished coding before the items disappeared, or they may have lost focus or had their minds wander during the long learning process (Zhao et al., 2020). This could have led to lower test scores.

There are limitations of this study that need to be addressed. First, as has been well documented, learning under multimedia condition is text-oriented (Makransky et al., 2019), and people tend to spend significantly more time on the text to obtain information effectively. In the coding stage, the more effective an object can obtain the learner’s attention resources, the less attention resources will be paid to other objects with lower saliency (Nardo et al., 2014). Cognitive system has limited resource and learners may inhibit the processing and understanding of other subordinate information by focusing too much on the marked content. That is, the prominent label attracts the learner’s attention and reduces the learner’s processing of the text. Thus, in future research, we can consider matching the color of the label with the color of the corresponding text, so that learners can quickly capture key information, and organize and integrate the text content to build a coherent mental representation. Second, our research, like most previous studies, only examines the impact of cue label attributes on people with low domain knowledge. Extending to more diverse group will promote the educational and practical significance of the research.

## Conclusion

Based on this study, the following conclusions can be drawn. (1) Cues promote learners’ memorization of content. Performance was better on the retention test under the large label condition. (2) Cues act as attentional guides. Learners eyes entered the cue area more frequently. (3) Although the combination of size and color shows a certain cue advantage in eye movement indicators, that is, it guides the learner’s attention, this cue advantage is not shown in academic performance. The mechanism of this result requires further exploration.

## Data Availability Statement

The original contributions presented in the study are included in the article/supplementary material, further inquiries can be directed to the corresponding author.

## Ethics Statement

The studies involving human participants were reviewed and approved by Fujian Normal University. The patients/participants provided their written informed consent to participate in this study.

## Author Contributions

JH and JZ developed the study concept and contributed to the study design. JH performed testing, data collection, data analysis, and interpretation under the supervision of JZ and drafted the manuscript. JZ provided critical revisions. All authors contributed to the article and approved the submitted version.

## Funding

This work was supported by the Key Research Institute of Humanities and Social Sciences, Ministry of Education, China (Grants 16JJD190004).

## Conflict of Interest

The authors declare that the research was conducted in the absence of any commercial or financial relationships that could be construed as a potential conflict of interest.

## Publisher’s Note

All claims expressed in this article are solely those of the authors and do not necessarily represent those of their affiliated organizations, or those of the publisher, the editors and the reviewers. Any product that may be evaluated in this article, or claim that may be made by its manufacturer, is not guaranteed or endorsed by the publisher.
